# Dataset and ANFIS model prediction of the performance of graphene nano-LPG in domestic refrigerator system

**DOI:** 10.1016/j.dib.2022.108548

**Published:** 2022-08-27

**Authors:** T.O. Babarinde, D.M. Madyira

**Affiliations:** a Department of Mechanical Engineering Science, University of Johannesburg, Johannesburg, South Africa; b Process, Energy and Environmental Technology Station (PEETS), University of Johannesburg, Johannesburg, South Africa

**Keywords:** Nanolubricant, LPG, Power consumption, Cooling capacity, COP, Experimental data, ANFIS training data, ANFIS testing data

## Abstract

LPG's steady-state performance in a base lubricant and a graphene nanolubricant was investigated in this study. Step-by-step processes and procedures for preparing graphene nanolubricant concentrations and replacing them for the base lubricant in a domestic refrigerator system were presented as the measuring devices necessary and their uncertainties. The experimental dataset and the training and testing datasets for Adaptive Neuro-fuzzy Inference System. (ANFIS) are available. The use of an ANFIS approach model to forecast graphene nanolubricant performance in a domestic refrigerator is described. The Root Mean Square Error (RMSE), Mean Absolute Deviation (MAD) and Mean Absolute Percentage Error (MAPE) are also available as statistical performance indicators for the ANFIS model prediction.

**Specifications Table**

**Table:** 

Subject:	Mechanical Engineering
Specific subject area"	Nanomaterials and Energy efficiency
Type of data:	Figure, Text file, Tables
How data were acquired:	Experimentation and mathematical analyses were used to obtain the data. The thermocouples, pressure gauges, and mass flow metre, as well as an electronic weighing scale for measuring nanoparticles and mass charge of the LPG refrigerant, were used as measuring instruments. The performance prediction of the Adaptive Neuro-Fuzzy inference system (ANFIS) model was developed on a computer running MATLAB software
Data format:	Raw, Analysed
Description of data collection:	Temperatures were measured at the inlet and exit of components of the refrigerator during the experiment. In addition, the pressures at the inlet and exit of the compressor were measured. Experimental data was first collected at 27 °C, and the data was captured by measuring instruments at 30 min intervals for 5 h. In ensuring the accuracy of the data the experiment was repeated 5 times. In the ANFIS, 70% of the experimental data is put to use for training.
Data source location:	Department of Mechanical Engineering Science, University of Johannesburg, South Africa
Data accessibility:	DOI:10.17632/9yc82m6yw5.1
Related research article:	T. O. Babarinde et al., “Improving the performance of LPG with graphene- nanolubricant in a domestic refrigerator: an artificial intelligence approach Improving the performance of LPG with graphene-nanolubricant in a domestic refrigerator: an artificial intelligence approach,” 2021. https://doi:10.1080/01430750.2021.1914160.

## Value of the Data

• The data allows for sizing and design of energy-efficient nano-LPG refrigerator system energy; however, there is an absence of real raw experimental data results showing a significant difference between predicted and experimental values. This experimental data provides expected variation data, and ANFIS predicted values that can be used to design and size a nano-LPG refrigerator system.
• The data contains steps for analysing collected data, a mathematical modelling for the performance of nano-LPG in refrigerator system is also presented as well as mathematical modelling for the performance of nano-LPG that can be used by refrigeration researchers and technicians to replace base refrigerant and lubricant with nano LPG in refrigerator systems.
• The data from the ANFIS model can be utilized to train and test artificial intelligence models for forecasting the performance of nano-LPG in household refrigerator systems.
• The results show that LPG/graphene-nanolubricant concentrations may be optimized in a household refrigerator utilizing LPG and that the method can be applied to various hydrocarbon refrigerants.

## 1 Data Description

**Fig. 1:**
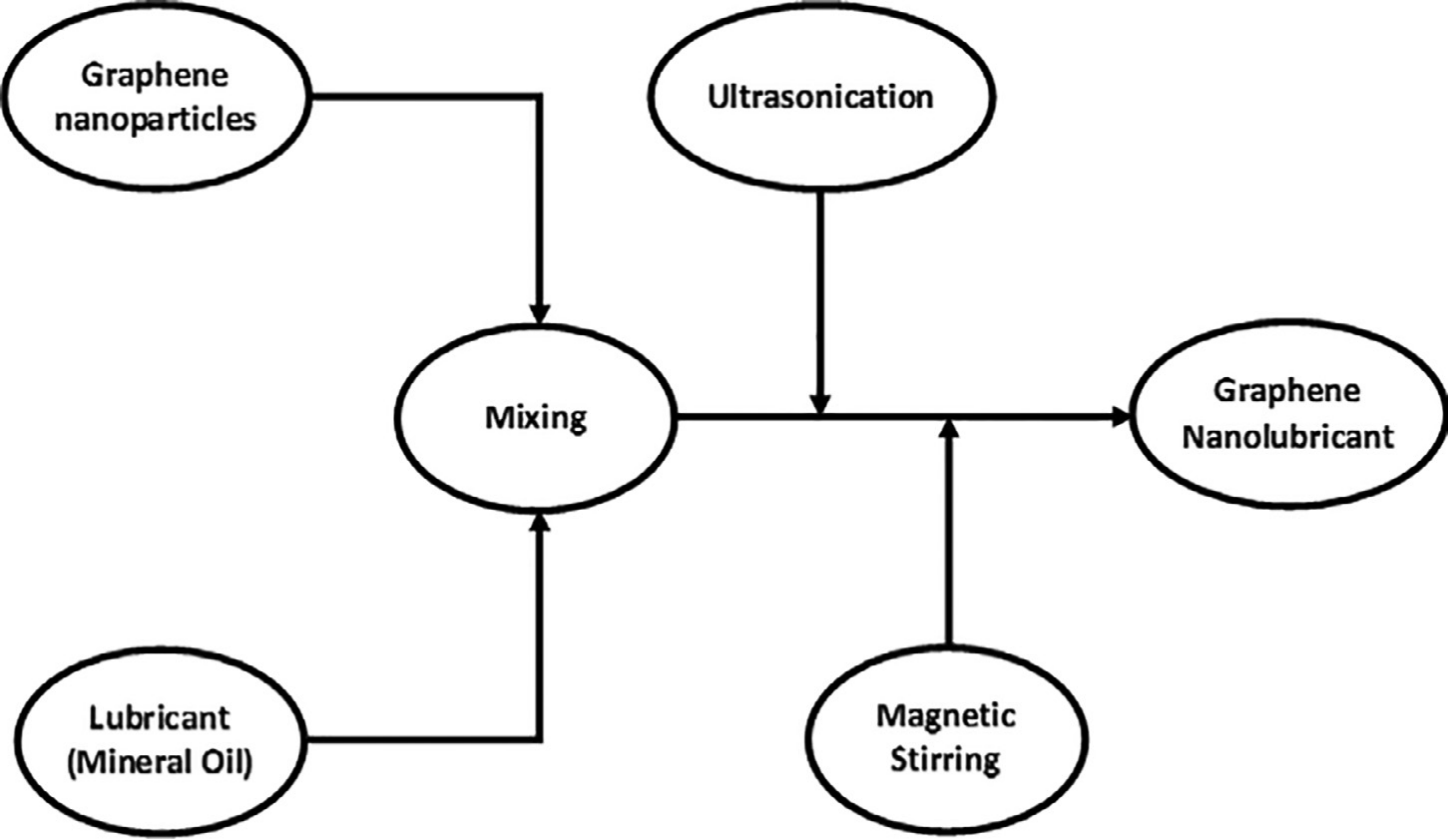
The graphene-nanolubricant flowchart.

**Fig. 2:**
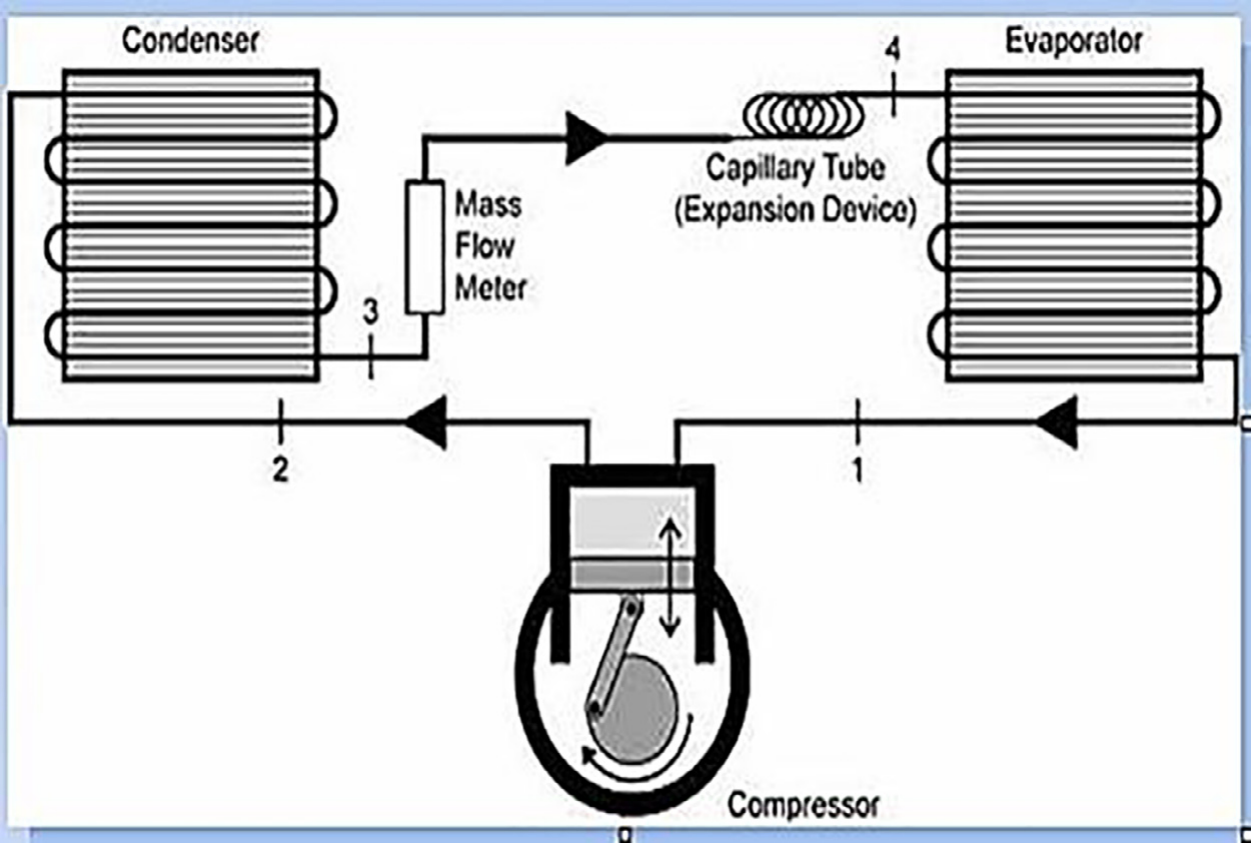
The experimental setup.

**Fig. 3:**
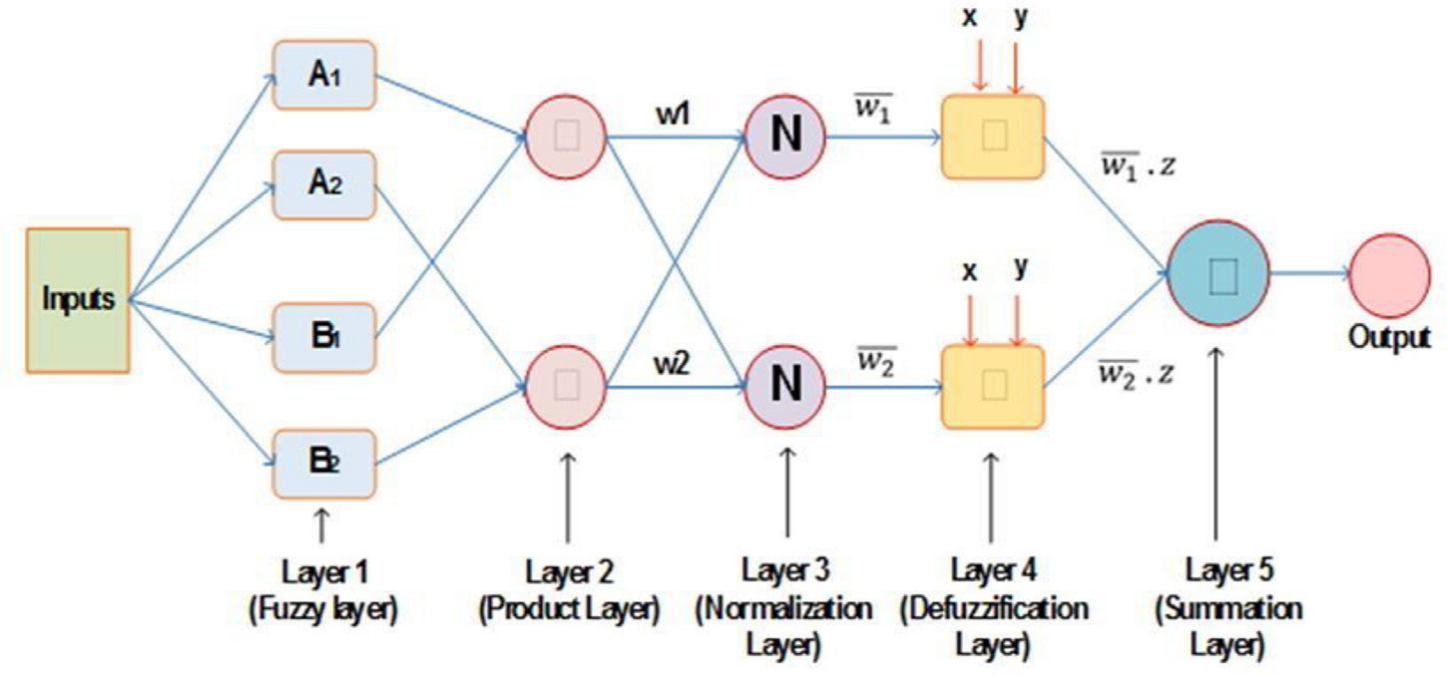
ANFIS architecture [2].

The experimental setup, as well as the data set from the graphene nano-LPG experiment, are explained. The graphene-nanolubricant is prepared in two steps, as presented in Fig. 1. The experimental setup showing the refrigerator component's inlet and exit as well as the test point where each data point is collected, are depicted in Fig. 2. The ANFIS architecture of the system's performance prediction showing the input and output data values is depicted in Fig. 3 [1]. Table 1 shows the data set that was obtained from various test points of the experiment, which includes graphene-nanolubricant -concentration (g/L), LPG mass charge (g), temperature (^C^) at the condenser, evaporator, and compressor inlet and exit pressure (KPa) and mass-flow (kg/s) of the LPG. Table 2 shows the LPG mass charge and nanolubricant concentration together with the enthalpy (kJ/kg) of the evaporator, compressor, and condenser and the LPG mass flow rate. The mass flow and nanolubricant data set are shown in Table 3 as well as the system's performance which includes cooling capacity (W), power consumption (W), and coefficient of performance. Table 4 presents the experimental data set for the performance of the system, which includes the training and testing data sets. Table 5 displays the statistical analysis of the predicted dataset, including RMSE, MAD, and MAPE as well as the number of iterations.

**Table 1:** Data set of temperatures and pressures of the system in steady-state.

S/N	Mass (g)	Nc (g/L)	Time (min)	T_1_ Evap. (°C)	T_3_ Cond. (°C)	P_2_ (kPa)	ḿref (g/s)
1	50	0	210	-9	42	788.3	0.70
2	50	0.2	120	-14	35	653.2	0.81
3	50	0.4	150	-10	41	767.8	0.78
4	50	0.6	180	-9	42	788.3	0.75
5	60	0	150	-4	50	967.2	0.65
6	60	0.2	150	-12	38	708.8	0.78
7	60	0.4	180	-12	36	671.3	0.73
8	60	0.6	210	-7	45	852.2	0.7
9	70	0	240	1	57	1459.8	0.57
10	70	0.2	180	-8	44	830.5	0.71
11	70	0.4	210	-5	48	920.0	0.67
12	70	0.6	240	-3	51	991.6	0.63

**Table 2:** Experiment data on the system's enthalpy at a steady state.

Mass (g)	Nc (g/L)	h_1_ (kJ/kg)	h_2_ (kJ/kg)	h_3_ (kJ/kg)	ḿref (g/s)
50	0	571.9	690.7	302.1	0.7
50	0.2	565.3	647.3	286.1	0.81
50	0.4	570.5	670.5	316.5	0.78
50	0.6	571.9	676.5	314.7	0.75
60	0	578.4	707.6	327.0	0.65
60	0.2	567.9	655.1	297.5	0.78
60	0.4	567.9	659.7	273.2	0.73
60	0.6	574.5	690.2	325.7	0.7
70	0	584.9	739.3	349.8	0.57
70	0.2	573.1	680.9	311.6	0.71
70	0.4	577.1	696.2	325.7	0.67
70	0.6	579.7	713.0	331.1	0.63

**Table 3:** Data from experiments on the system's steady-state performance.

Mass (g)	Nc (g/L)	QE (W)	WC (W)	COP
50	0	188.8	83.2	2.3
50	0.2	226.1	66.5	3.4
50	0.4	198.1	78.0	2.5
50	0.6	192.9	78.5	2.5
60	0	163.4	84.0	2.0
60	0.2	210.9	68.0	3.1
60	0.4	215.1	67.0	3.2
60	0.6	174.1	81.0	2.1
70	0	134.0	88.0	1.5
70	0.2	185.7	76.5	2.4
70	0.4	168.4	79.8	2.1
70	0.6	156.6	84.0	1.9

**Table 4:** Data showing the system's training and testing datasets at a steady state.

Experimental (WC(W))		ANFIS Predicted (WC(W))	Experimental (QE (W))		ANFIS predicted (QE (W))	Experimental COP		ANFIS predicted COP
84		84.00001	156.6		156.6000	1.9		1.8999
81	Training	80.99998	174.1	Training	174.0999	2.1	Training	2.0999
76.5	data	76.49996	185.7	data	185.7001	2.4	data	2.4000
66.5		66.5001	226.1		226.0998	3.4		3.3998
84.0		84.0001	163.4		163.3999	2.0		1.9998
68.0		67.99999	210.9		210.9122	3.1		3.1000
67.0		67.00001	215.1		215.0998	3.2		3.2000
83.2		83.20001	188.8		188.7999	2.3		2.2999
78.0	Testing data	82.56821	198.1	Testing data	191.4103	2.5	Testing data	2.2825
79.8		82.35476	168.4		153.5329	2.1		1.8628
88.0		87.28389	134.0		163.5083	1.5		1.9743
78.5		83.19999	192.9		168.5266	2.5		2.3000

**Table 5:** The ANFIS models' performance evaluation.

Performance metrics	Power Consumption (WC(W))		Cooling Capacity (QE (W))		COP	
	Train	Test	Train	Test	Train	Test
RMSE	1.67E-05	3.5355	1.00E-04	20.8002	2.62E-07	0.3035
MAD	1.14E-05	1.8574	6.12E-05	16.8069	2.02E-07	0.2597
MAPE (%)	1.33E-05	3.9648	4.34E-05	11.7155	8.99E-06	14.9032
No of Iterations	8		8		8	

## 2 Experimental Design, Materials and Methods

The test rig in this experiment was a regular refrigerator. The system was evacuated using a vacuum flusher. On a digital scale, graphene nanoparticles were weighed, and 0.2 g/L, 0.4 g/L, and 0.6 g/L samples were prepared to be evaluated in 50 g, 60 g, and 70 g LPG refrigerant charges. The nanolubricant sample was agitated using an ultrasonic oscillator as shown in Fig. 1 [1].

The LPG refrigerant was added to the system *via* an electronic charging device. The temperature at the inflow and outflow of various system components was measured using four K-type thermocouples. To measure the compressor's suction and discharge pressures, the pressure gauges were also connected to the suction and discharge ports. The experimental test rig is shown in Fig. 2.

Table 1 displays the characteristic condition ranges of the experiment and also the measurement uncertainty. Temperature and pressure values were measured five times, with a 30 min gap between each. The uncertainties of the measurement devices used, which include a thermocouple, a pressure gauge, and a power meter, are +  3%, + 1%, and + 1%, respectively. The temperature and pressure data outputs from the Ref-prop, version 9.0, were used to calculate the refrigerant's enthalpy and entropy. These values were used to calculate the coefficient of performance (COP), cooling capacity, and power consumption. The performance evaluation for the experimental outcome was determined using the Eqs. (1)–(3) [3].

Eq. (1) was used to compute the cooling capacity ($Q_{E}$)

(1) $$Q_{E}=m_{ref.}\left(h_{1}-h_{4}\right)$$

The enthalpies at the evaporator's exit and inlet are indicated by $h_{1}$ and $h_{4}$.

Eq. (2) is used to define the system's compressor power input ($W_{C}$)

(2) $$W_{C}=m_{ref.}\left(h_{2}-h_{1}\right)$$

The enthalpy at the compressor's inlet and outlet are represented by $h_{1}$ and $h_{2}$. Eq. (3) is used to compute the COP.

(3) $$COP=\;\frac{Q_{E}}{W_{C}}$$

### 2.1 ANFIS Modeling

The ANFIS modeling technique is a five-layered feedforward network that combines ANN with fuzzy logic functions to build a hybrid model capable of function fitting and effective performance in systems with imprecision and uncertainty. Fig. 3 illustrates the model architecture. In ANFIS, all nodes are adaptive in the first and fourth layers, but non-adaptive nodes exist in the other layers. Takagi Sugeno's fuzzy inference system is used in this model (TSFIS). An if-then principle relates the antecedent and consequent in the following way:

ANFIS is a five-layer feed-forward network that combines ANN with Fuzzy logic technologies to create a hybrid model capable of practical fit and efficient performance in systems with imprecision and uncertainty. All nodes in ANFIS are adaptive in the first and fourth levels, however, non-adaptive nodes exist in the other layers. Takagi Sugeno's Fuzzy Inference System is used in the model (TSFIS). Evaporator temperature (T_1_), condensing temperature (T_3_), refrigerant mass charge, and nanolubricant concentration are the input data for these models (Nc). As previously indicated, 70% of the data was utilized for training and 30% of the data was also used for model testing.

The ANFIS MATLAB model program can be found in the supplementary file, which is available online. The ANFIS model's rules are based on an if-then concept that ties the antecedent to the result as follows:

(4) $$\text{Condition}\;1:\;\text{If}\;X_{1}\;\text{is}\;A_{1}\;\text{AND}\;X_{2}\;\text{is}\;B_{1}\;\text{then}\;f_{1}=p_{1}X_{1}+q_{1}X_{2}+r_{1}$$

(5) $$\text{Condition}\;2:\;\text{If}\;X_{1}\;\text{is}\;A_{2}\;\text{AND}\;X_{2}\;\text{is}\;B_{2}\;\text{then}\;f_{2}=p_{2}X_{1}+q_{2}X_{2}+r_{2}$$

$X_{1}$ and $X_{2}$ are model inputs, $A_{1}$ and $A_{2}$, $B_{2}$ and $B_{2}$ are fuzzy sets, while p, q and r are node parameters.

Fig. 3 depicts the ANFIS structure model. The following is how each layer is parameterized:

Layer 1: This layer is made up of adaptive nodes that have a fuzzy membership function and whose output function is calculated as follows:

(6) $$O_{j}^{1}=\mu_{\text{Aj}}\left(I_{1}\right),j=1,2$$

(7) $$O_{j}^{1}=\mu_{\text{Bj}}\left(I_{2}\right),j=1,2$$

Layer 2: This layer's nodes are nonadaptive, and each rule's firing strength is calculated using a multiplicative operator corresponding to Eq. (11).

(8) $$O_{j}^{2}=w_{j}=\mu_{\text{Aj}}\left(I_{1}\right).\mu_{\text{Bj}}\left(I_{2}\right),j\;=1,2$$

Layer 3: According to [4], the nodes in this layer are also set using normalisation of the firing strength, with the *j*th node performing through using the ratio of its firing strength to the collective of all firing strengths from all rules:

(9) $$O_{j}^{3}={\bar{w}}_{j}=\frac{w_{j}}{w_{1}+w_{2}}\;j\;=1,2$$

Layer 4: This layer's nodes are adaptive, and it defuzzer the data. Eq. (13) is used to express the influence of the jth rule on the layer output, and the node parameters are depicted by $p_{i}$, $q_{j}$, and $r_{j}$.

(10) $$O_{j}^{4}={\bar{w}}_{i}\left(p_{j}I_{1}+q_{j}I_{2}+r_{j}\right)={\bar{w}}_{i}z_{j}$$

Layer 5: This layer comprises nonadoptive nodes that use a summing function to combine all of the incoming signals from the preceding layer [5].

(11) $$O_{j}^{5}=\sum_{j}{\bar{w}}_{i}z_{j}=\;\frac{\sum_{j}w_{i}z_{j}}{\sum_{j}w_{i}}$$

The best network was put to the test withhold-out data. The model outputs were compared to the ANFIS predicted values and statistical performance metrics such as Root Mean Square Error (RMSE), Mean Absolute Percentage Error (MAPE), and Mean Absolute Deviation (MAD) (MAD). The following measures were calculated using Eqs. (12) to (14) [1].

Root Mean Square Error (RMSE)

(12) $$RMSE=\sqrt{\frac{\sum_{K=1}^{N}{\left[y_{k}-{\hat{y}}_{k}\right]}^{2}}{N}}$$

Mean Absolute Deviation (MAD)

(13) $$MAD=\frac{1}{N}\sum_{K=1}^{N}\left|y_{k}-\bar{y}\right|$$

Mean Absolute Percentage Error (MAPE)

(14) $$MAPE=\frac{1}{N}\sum_{K=1}^{N}\left|\frac{y_{k}-{\hat{y}}_{k}}{y_{k}}\right|X\;100\%$$

where, $y_{k}$ is the actual value,$\;{\hat{y}}_{k}$ denotes the anticipated value, and $\bar{y}$ denotes the observed mean.

## Declaration of Competing Interest

None.

## Data Availability

Dataset of graphene nano-liquified Petroleum Gas (LPG) performance in a domestic refrigerator system (Original data) (Mendeley Data).
